# AGE AND STRESSOR-RELATED NEGATIVE AFFECT IN OLDER ADULTS: CROSS-SECTIONAL AND LONGITUDINAL EVIDENCE

**DOI:** 10.1093/geroni/igad104.1403

**Published:** 2023-12-21

**Authors:** Giselle Ferguson, Daisy Zavala, Samantha Corley, Stacey Scott

**Affiliations:** Stony Brook University, The State University of New York, Stony Brook, New York, United States; Stony Brook University, Stony Brook, California, United States; Stony Brook University, Stony Brook, New York, United States; Stony Brook University, Stony Brook, New York, United States

## Abstract

Most daily stress studies show stressor-related increases in Negative affect (NA), but older adults may show smaller increases due to emotion-regulation skills. However, most of this work is based upon single burst diary or ecological momentary assessment (EMA) studies which are functionally cross-sectional age comparisons; longitudinal analyses are needed to reveal whether individuals change as they age. In this secondary analysis of data from a measurement burst study of older adults in Bronx, NY (N=305; Mage=76.98), we examined how age interacted cross-sectionally and longitudinally with the effect of stressors on NA. Between 2017- (pre-pandemic) 2020, participants completed up to three 14-day EMA bursts. Five times daily, they reported current NA and whether a stressor had occurred since the last assessment. In a 4-level multilevel model accounting for demographics, Neuroticism, and stressors, NA was significantly higher with reports of stressors, including at the moment-level, day-level, and person-level. Regarding aging’s role in average NA for non-stressor moments, there were not significant cross-sectional age differences, but longitudinally across three bursts, NA increased significantly within-persons. We did not find evidence for cross-sectional age differences nor longitudinal change in the stressor-NA slope. Although neither cross-sectional age differences nor longitudinal change was found in the stressor-NA slope, we found significant longitudinal increases in non-stressor NA as individuals aged. Our results illustrate the value of measurement burst designs for disentangling differences between cross-sectional and longitudinal effects in daily processes. Further research on aging-related differences in affect and stress would benefit from these approaches.

